# Drug Safety Profiles of Geriatric Patients Referred to Consultation Psychiatry in the Emergency Department—A Retrospective Cohort Study

**DOI:** 10.1177/08919887221149158

**Published:** 2023-01-02

**Authors:** Martin Schulze Westhoff, Sebastian Schröder, Johannes Heck, Torben Brod, Marcel Winkelmann, Stefan Bleich, Helge Frieling, Kirsten Jahn, Felix Wedegärtner, Adrian Groh

**Affiliations:** 1 Department of Psychiatry, Social Psychiatry and Psychotherapy, 9177 Hannover Medical School, Hannover, Germany; 2 Institute for Clinical Pharmacology, 9177 Hannover Medical School, Hannover, Germany; 3 Emergency Department, 9177 Hannover Medical School, Hannover, Germany; 4 Trauma Department, 9177 Hannover Medical School, Hannover, Germany

**Keywords:** consultation psychiatry, emergency department, drug safety, potentially inappropriate medications, elderly

## Abstract

**Objective:**

Geriatric patients account for a significant proportion of the collective treated by psychiatric consultation service in hospitals. In the Emergency Department (ED), psychotropic drugs are frequently recommended, notwithstanding their extensive side-effect profiles. This study sought to investigate medication safety of geriatric patients referred to psychiatric consultation service in the ED.

**Methods:**

Medication lists of 60 patients from the general internal medicine and trauma surgery EDs referred to psychiatric consultation service were analyzed. Utilizing PRISCUS list and Fit fOR The Aged (FORTA) classification, prescriptions of potentially inappropriate medications (PIMs) were assessed.

**Results:**

84 drugs were newly prescribed following psychiatric consultations. The total number of drugs per patient was 5.4 ± 4.2 before psychiatric consultation and 6.5 ± 4.2 thereafter (*p* < .001). 22.6 % of the newly recommended drugs were PIMs according to the PRISCUS list, while 54.8 % were designated as therapeutic alternatives to PIMs. 54.8 % and 20.2 % of the newly recommended drugs were FORTA category C and D drugs, respectively. An average of 1.2 ± 1.7 drug–drug interactions (DDIs) existed before psychiatric consultation and 1.3 ± 1.9 DDIs thereafter (*p* = .08).

**Conclusion:**

The majority of newly recommended drugs by psychiatric consultation service in the ED were designated as suitable therapeutic alternatives to PIMs according to the PRISCUS list, but had comparatively unfavorable ratings according to the FORTA classification, demonstrating discrepancies between these two PIM classification systems. Physicians delivering psychiatric consultation services in the ED should not solely rely on one PIM classification system.

## Introduction

For up to 6 % of all inpatients undergoing treatment for a general medical or surgical condition, a psychiatric consultation service is contacted during their hospital stay.^1^ Diagnostic classification of the psychopathology by the consulted psychiatrist often does not correspond to the diagnosis originally assumed by the treating specialist (e.g., internist, surgeon).^2^ It has been shown that consultation-liaison psychiatry in hospitals significantly contributes to reduction of health-economic costs, shorter hospital stays, and better patient-related outcomes.^3,4^ Previous studies demonstrated that about 50 % of psychiatric consultations involve geriatric patients, and that the proportion of geriatric patients has constantly increased in recent years.^5^

Geriatric patients are particularly prone to adverse drug reactions (ADRs) due to physiologically altered pharmacodynamic and pharmacokinetic properties and multimorbidity.^6,7^ In many cases, elderly patients exhibit polypharmacy, the simultaneous intake of five or more drugs.^8,9^ Older age and polypharmacy are associated with drug–drug interactions (DDIs) and prescription of potentially inappropriate medications for the elderly (PIMs).^10^ The presence of DDIs and PIMs increases the risk of ADRs.^8^ Due to numerous somatic comorbidities, geriatric psychiatric patients represent a high-risk population for the occurrence of ADRs.^11^

Previous studies showed that pharmacological recommendations are made in up to 50 % of psychiatric consultations.^12,13^ Particularly in the Emergency Department (ED), critically ill geriatric patients with acute psychiatric disorders are often treated with psychotropic drugs that display an extensive side-effect profile.^14^ This study sought to assess medication safety of geriatric patients in the context of psychiatric consultation service in the ED.

## Methods

### Ethics Approval

This study was approved by the Ethics Committee of Hannover Medical School (No. 10505_BO_K_2022) and adheres to the Declaration of Helsinki (1964) and its later amendments (current version from 2013).

### Eligibility Criteria

Patients were enrolled in the study (i) if they were ≥ 65 years of age, (ii) if they were treated in the general internal medicine ED or trauma surgery ED of Hannover Medical School between January 2017 and April 2022, (iii) if they were referred to psychiatric consultation service by the treating internist(s) or surgeon(s) with the request to recommend a change of medication, and (iv) if they or their legal representative had provided written informed consent that patient-related data be used for clinical research. Hannover Medical School is a large university hospital and tertiary care center in northern Germany. The general internal medicine and trauma surgery EDs are frequented by about 25,000 patients per year (136,003 patients during the study period). During the study period, psychiatric consultation service was involved in the treatment of 1,263 patients (937 patients of the general internal medicine ED and 326 of the trauma surgery ED).

### Medication Chart Reviews, PIM Classification Systems, Drug Interaction Checks, and Demographic Characteristics

Medication charts of enrolled patients were analyzed before and after psychiatric consultation by an interdisciplinary team of experts in psychiatry and clinical pharmacology. Recommended changes of medication (e.g., newly prescribed or discontinued drugs) were recorded for each patient. All newly prescribed or discontinued drugs (both regularly taken drugs and *pro re nata* drugs) were assessed with the aid of the PRISCUS list (*priscus* (Latin), ancient, venerable) and the Fit fOR The Aged (FORTA) classification.

The PRISCUS list tabulates a total of 83 drugs considered as PIMs.^15^ The PRISCUS list is tailored to the German pharmaceutical market and applies to people ≥ 65 years. Besides listing PIMs, the PRISCUS list offers recommendations of suitable pharmacological alternatives for the treatment of elderly people. For the purpose of this study, we categorized the drug prescriptions in our study population as PIMs (according to the PRISCUS list), non-PIMs (i.e., drugs not listed as PIMs on the PRISCUS list), and suitable therapeutic alternatives to PIMs (according to the PRISCUS list).

The FORTA classification categorizes drugs into four classes (i.e., A to D), based on their therapeutic indications: A = indispensable drugs in the pharmacological treatment of elderly people; B = drugs with proven or obvious efficacy in elderly people; C = drugs with questionable efficacy–safety profiles in elderly people; D = drugs that should be avoided in elderly people.^16^ In this study, drugs not mentioned in the FORTA classification were classified as “Not labelled.” Similar to the PRISCUS list, the FORTA classification was developed in Germany and applies to people ≥ 65 years.

Drug interaction checks were performed for all medication lists before and after psychiatric consultation with the tool “Medibox” of the electronic drug information system AiDKlinik® (Arzneimittel-Informations-Dienste, Dosing GmbH, Heidelberg, Germany). Only DDIs that were classified as “moderate,” “severe” or “contraindicated combination” by AiDKlinik® were considered for statistical analysis.

Demographic characteristics—i.e., age, sex, and International Statistical Classification of Diseases and Related Health Problems 10th Revision (ICD-10) diagnoses—were retrieved from the patient records.

### Statistical Analysis

Quantitative variables are depicted as means ± standard deviations. For categorical variables, absolute and relative frequencies were calculated. All statistical analyses were performed with IBM® SPSS® Statistics for Windows, version 28 (Armonk, New York, USA). *P* values < 0.05 were considered statistically significant. Testing for normal distribution was carried out by utilization of the Shapiro–Wilk test. For comparison of the number of drugs and the number of DDIs before and after psychiatric consultation Wilcoxon signed rank tests were applied.

## Results

### Study Population and Drug Recommendations

**Figure 1.**
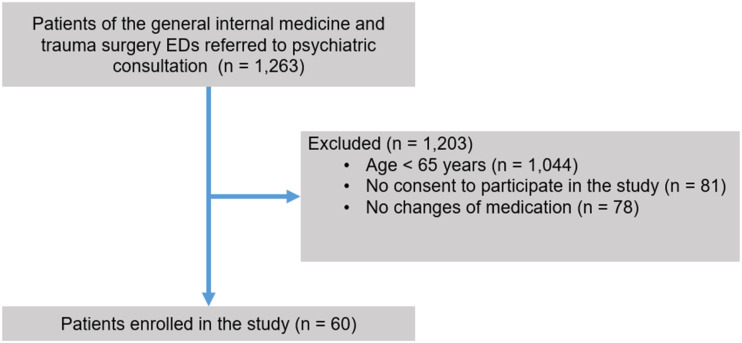
Flow of participants. Patients of the general internal medicine and trauma surgery EDs were eligible for inclusion in the study if they were referred to psychiatric consultation service between January 2017 and April 2022 with the request of medication change. Patients needed to be at least 65 years of age and they or their legal representative had to provide written informed consent. ED denotes Emergency Department.

60 of 1,263 screened patients fulfilled the eligibility criteria and were enrolled in the study (Figure 1). The main reason for exclusion was age < 65 years (1,044 of 1,263 screened patients). The mean age of the study population (n = 60) was 74.3 ± 7.6 years and 50 % (30/60) of the patients were female (Table 1). Delirium was the most frequent psychiatric diagnosis in the study population (33.3 %; 20/60), followed by depression (28.3 %; 17/60) and dementia (23.3 %; 14/60). The most prevalent somatic comorbidity was arterial hypertension, which affected half (50 %; 30/60) of the study population. The main reason for psychiatric consultation was agitation (31.7 %, 19/60), followed by suicidality and self-harming behavior (28.3 %, 17/60).

**Table 1.** Characteristics of the Study Population (n = 60). The Mean Age ± Standard Deviation of the Study Population was 74.3 ± 7.6 Years.

Variables	n	%
Sex		
Female	30	50.0
Male	30	50.0
Psychiatric diagnoses^a^		
Depression^b^	17	28.3
Bipolar affective disorder^c^	1	1.7
Schizophrenia or schizophreniform disorder^d^	6	10
Mental and behavioral disorder due to use of alcohol, tobacco, or sedatives or hypnotics^e^	14	23.3
Dementia^f^	14	23.3
Delirium^g^	20	33.3
Other psychiatric disorder(s)	14	23.3
Somatic diagnoses^a^		
Arterial hypertension	30	50.0
Coronary heart disease	16	26.6
Chronic heart failure	13	21.6
Atrial fibrillation	9	15.0
Status post stroke	3	5.0
Type-2 diabetes mellitus	13	21.6
Chronic obstructive pulmonary disease	6	10.0
Hypothyroidism	7	11.7
Urinary tract infection	7	11.7
Other somatic disorder(s)	57	95.0
Main reasons for psychiatric consultation		
Agitation	19	31.7
Suicidality and self-harming behavior	17	28.3
Substance-related (e.g., withdrawal symptoms)	12	20.0
Acute psychosis	12	20.0

^a^Patients could have more than one diagnosis.

^b^ICD-10 F32, F33.

^c^ICD-10 F31.

^d^ICD-10 F06.2, F2X.

^e^ICD-10 F10, F13, F17.

^f^ICD-10 F00, F01, F02, F03.

^g^ICD-10 F05. ICD-10 denotes International Statistical Classification of Diseases and Related Health Problems 10th Revision.

In total, 108 pharmacological recommendations were made by psychiatric consultation service. In 84 cases, the start of a new medication was proposed, whereas in 22 cases drug discontinuation or dose reduction were recommended. Two recommendations concerned the timing of drug intake.

Patients received an average of 5.4 ± 4.2 drugs before psychiatric consultation and 6.5 ± 4.2 drugs afterwards (*p* < .001). The most frequently newly recommended drugs were pipamperone (19.0 %; 16/84), risperidone (15.5 %; 13/84), and oxazepam (13.1 %; 11/84). 88.1 % (74/84) of the newly recommended drugs, with the exception of antidepressants and certain antipsychotic drugs, were intended for use in the acute situation in the ED or for short-term use. Recommendations regarding dose reduction or discontinuation of medication frequently involved olanzapine, opipramol, valproate, citalopram, and lorazepam (9.1 % (2/22) each).

### Potentially Inappropriate Medications for Older People According to the PRISCUS List

**Figure 2.**
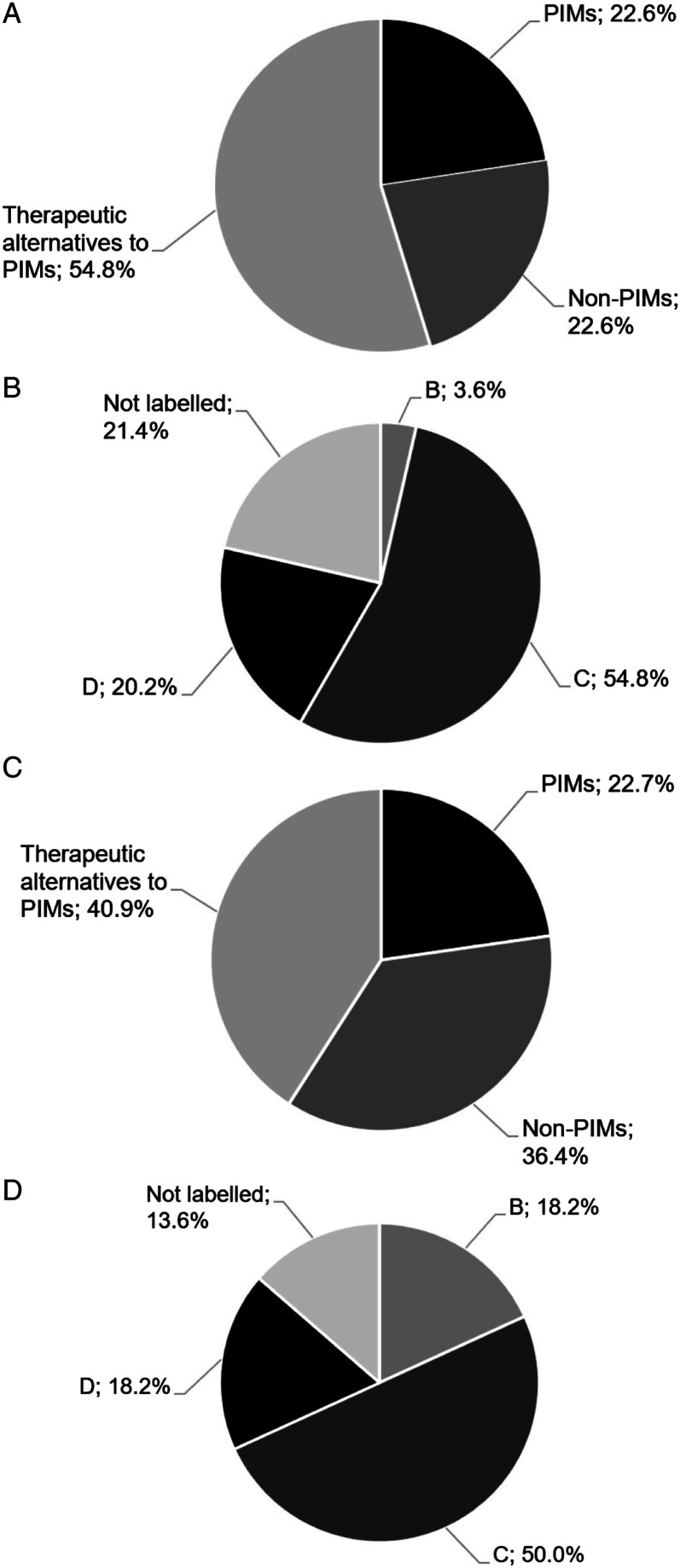
Categorization of newly recommended drugs (n = 84; **A, B**) and discontinued drugs (n = 22; **C, D**) in the study population according to the PRISCUS list and the FORTA classification. **A** Categorization of newly recommended drugs according to the PRISCUS list. **B** Categorization of newly recommended drugs according to the FORTA classification. **C** Categorization of discontinued drugs according to the PRISCUS list. **D** Categorization of discontinued drugs according to the FORTA classification. PIM denotes potentially inappropriate medication for elderly people (i.e., ≥ 65 years of age), FORTA Fit fOR The Aged. FORTA classes A to D are defined as follows: A = indispensable drugs in the pharmacological treatment of elderly people; B = drugs with proven or obvious efficacy in elderly people; C = drugs with questionable efficacy–safety profiles in elderly people; and D = drugs that should be avoided in elderly people.

22.6 % (19/84) of the newly prescribed drugs were PIMs according to the PRISCUS list (PRISCUS-PIMs), whereas 54.8 % (46/84) were listed as suitable therapeutic alternatives to PIMs according to the PRISCUS list (Figure 2A**)**. The most frequently recommended PRISCUS-PIMs were oxazepam > 60 mg/d (47.4 %; 9/19), lorazepam > 2 mg/d (21.1 %; 4/19), and haloperidol > 2 mg/d (10.5 %; 2/19) (Table 2). In contrast, pipamperone (34.8 %; 16/46), risperidone (28.3 %; 13/46), and lorazepam ≤ 2 mg/d (8.7 %; 4/46) were the most frequently newly recommended drugs listed as suitable therapeutic alternatives to PIMs.

**Table 2.** Absolute and Relative Frequencies of Potentially Inappropriate Medications for Older People (i.e., ≥ 65 Years of Age) According to the PRISCUS List That Were Newly Prescribed in the Study Population.

Potentially inappropriate medication	n	%
All potentially inappropriate medications	19	100
Oxazepam (> 60 mg/d)	9	47.4
Lorazepam (> 2 mg/d)	4	21.1
Haloperidol (> 2 mg/d)	2	10.5
Levomepromazine	1	5.3
Clonidine	1	5.3
Clozapine	1	5.3

Recommendations for drug discontinuation or dose reduction (n = 22) involved PRISCUS-PIMs in 22.7 % (5/22), whereas 36.4 % (8/22) were not PRICUS-labelled and 40.9 % (9/22) were listed as suitable therapeutic alternatives to PIMs according to the PRISCUS list (Figure 2C).

Of note, 28.3 % (17/60) of all patients were affected by a new prescription of at least one PRISCUS-PIM by psychiatric consultation service, while 8.3 % (5/60) of patients had a PIM terminated following psychiatric consultation.

### Categorization of Drug Prescriptions According to the FORTA Classification

Of the newly recommended drugs, 0 %, 3.6 % (3/84), 54.8 % (46/84), and 20.2 % (17/84) were classified as FORTA category A, B, C, and D, respectively. 21.4 % (17/84) of the newly recommended drugs were not indexed in the FORTA classification (Figure 2B).

60 % (36/60) of all patients were affected by the new prescription of at least one FORTA C drug, and 25 % (15/60) of patients by at least one FORTA D drug. The most frequently newly prescribed FORTA C drugs were pipamperone (34.8 %; 16/46), risperidone (28.3 %; 13/46), and lorazepam (17.4 %; 8/46), whereas oxazepam (64.7 %; 11/17) and haloperidol (17.6 %; 3/17) were the most frequently newly prescribed drugs labelled as FORTA D (Table 3). Drugs recommended for dose reduction or discontinuation by the psychiatric consultation service were labelled as FORTA C in 50 % (11/22) of cases and FORTA B or D in 18.2 % (4/22) each (Figure 2D).

**Table 3.** Absolute and Relative Frequencies of FORTA Class C Drugs (i.e., Drugs with Questionable Efficacy–Safety Profiles in Elderly People) and FORTA Class D Drugs (i.e., Drugs that Should be Avoided in Elderly People) Newly Prescribed in the Study Population Following Psychiatric Consultation.

Drug	n	%
FORTA class C drugs	46	100
Pipamperone	16	34.8
Risperidone	13	28.3
Lorazepam	8	17.6
Melperone	3	6.5
Mirtazapine	3	6.5
Venlafaxine	1	2.2
Carbamazepine	1	2.2
Tilidine	1	2.2
		
FORTA class D drugs	17	100
Oxazepam	11	64.7
Haloperidol	3	17.6
Diazepam	1	5.9
Clozapine	1	5.9
Clonidine	1	5.9

FORTA denotes Fit fOR The Aged.

### Drug Interaction Checks

Drug interaction checks revealed 1.2 ± 1.7 DDIs per patient before psychiatric consultation and 1.3 ± 1.9 DDIs afterwards (*p* = .08). 43.3 % (26/60) of all patients were affected by at least one DDI before psychiatric consultation and 50 % (30/60) thereafter. Overall, there were 69 DDIs classified as “moderate,” “severe”, or “contraindicated combinations” in the study population before and 79 DDIs after psychiatric consultation. One contraindicated combination (i.e., combination of haloperidol and citalopram) was present in the study population, and discontinuation of this drug combination was recommended by psychiatric consultation service. Before psychiatric consultation, 18.8 % (13/69) of all DDIs were classified as “severe”; after psychiatric consultation this was the case in 19.0 % (15/79) of all DDIs.

Before psychiatric consultation the most frequent DDI categories were “increased bleeding risk” (21.7 %; 15/69), “pharmacodynamic antagonism” (18.8 %; 13/69), “central nervous system depressant effects” (13.0 %; 9/69), and “orthostatic hypotension” (13.0 %; 9/69).

After psychiatric consultation the most frequent DDI categories were “pharmacodynamic antagonism” (24.1 %; 19/79), “increased bleeding risk” (19.0 %; 15/79), and “orthostatic hypotension” (12.7 %; 10/79). The most frequent newly recommended drug combination with interaction potential was the combination of risperidone and pipamperone, accounting for 10.1 % (8/79) of all DDIs after psychiatric consultation. Psychotropic drugs most frequently involved in DDIs after psychiatric consultation—in addition to risperidone (16.5 %; 13/79) and pipamperone (10.1 %; 8/79)—were valproate (6.3 %; 5/79) and lorazepam (5.1 %; 4/79). Notably, haloperidol was involved in 20 % (3/15) of all DDIs rated as “severe.”

## Discussion

The present study investigated medication safety profiles of geriatric patients treated in EDs of internal medicine and trauma surgery of a large university hospital in Germany who were referred to psychiatric consultation service. The frequency of PIM prescriptions according to the PRISCUS list and the FORTA classification, as well as the number and severity of DDIs before and after psychiatric consultation were analyzed as indicators of the quality and adequacy of patients’ pharmacotherapy.

To the best of our knowledge, comparable studies examining medication safety in the context of psychiatric consultation service for geriatric patients in the ED do not exist to date. However, two recent studies also focused on geriatric inpatients who were referred to psychiatric consultation.^5,13^ Similar to these investigations, delirium, dementia, and depression were identified as the most common psychiatric diagnoses in our study. Furthermore, in terms of age, sex, and somatic comorbidities, our study population showed great similarities with those from previous analyses that focused on geriatric psychiatric inpatients^11,17^

Frequency and characteristics of PIM prescriptions have been quite extensively studied in both inpatient and outpatient settings.^18-20^ PIM prescription rates differed significantly, which can be attributed to different settings and study designs. In the context of the psychiatric consultation service, however, this question has not yet been investigated. Nevertheless, previous studies demonstrated that geriatric patients were prescribed fewer benzodiazepines and more atypical antipsychotics by psychiatric consultation service than younger comparison groups.^5,13^ This was explained by more frequent occurrence of neurocognitive disorders in advanced age, which are frequently treated symptomatically with atypical antipsychotics, and by a presumed reluctance of treating physicians to prescribe benzodiazepines in elderly patients due to pronounced side effects.^5,13^ In a large retrospective analysis, Hefner et al. found that 33.9 % of geriatric psychiatric inpatients were affected by the prescription of at least one PRISCUS-PIM.^11^ Seifert and colleagues showed that 5.7 % of all prescriptions on gerontopsychiatric wards involved PRISCUS-listed drugs.^17^ A large study from Taiwan detected that 32.4 % of all patients in the ED received at least one PRISCUS-listed drug.^21^ Dormann et al. identified a proportion of 16.6 % PRISCUS-PIMs among drug prescriptions in a German ED.^6^

In our study, 28.3 % of all patients were affected by new prescription of a PRISCUS-PIM. Overall, PRISCUS-PIMs accounted for 22.6 % of all new prescriptions, which was considerably higher than the 5.7 % of all prescriptions detected by Seifert et al.^17^ However, a comparison between the two studies is difficult since the Seifert et al. study referred exclusively to inpatients and also included medications for the treatment of somatic comorbidities. In addition, the comparatively high proportion of new prescriptions of therapeutic alternatives to PRISCUS-PIMs in our study and the fact that discontinuation of PRISCUS-PIMs was recommended in 8.3 % of all patients can be viewed as indicators of an adequate pharmacotherapy safety within the psychiatric consultation service at our hospital.

In contrast to the PRISCUS list, prescription of PIMs based on the FORTA classification has hardly been investigated. In two studies in the inpatient setting and in nursing homes, the proportion of patients receiving a PIM according to the FORTA classification was determined between 40 % and 55 %, which was significantly higher than the proportion of patients receiving at least one PRISCUS-PIM.^22,23^ In the present study, a proportion of 54.8 % and 20.2 % of FORTA C and FORTA D labelled medication recommendations were found. By contrast, among geriatric patients with Parkinson’s disease, Greten and colleagues found a proportion of 13.9 % FORTA C and 3.8 % FORTA D drugs among all prescribed drugs.^24^ Thus, according to the FORTA classification, significantly more PIMs appear to be recommended by psychiatric consultation service in the ED than in inpatient settings. Also, in our analysis, 60 % and 25 % of study participants were affected by at least one prescription of a FORTA C and FORTA D drug, respectively. Therefore, a high risk of ADRs and an overall unfavorable risk–benefit ratio of the pharmacological recommendations made by psychiatric consultation service for patients in the ED might be assumed, since, according to the FORTA classification, almost 75 % of newly commenced drugs should be discussed critically or avoided altogether. However, this finding must be counterbalanced with the fact that the recommendations of the FORTA classification concern 296 drugs and are thus significantly more comprehensive than those of the PRISCUS list, which only contains 83 agents, making a comparison of the two PIM classifications difficult.^15,16^ It should also be noted that both PIM classifications are not specifically designed for use in geriatric psychiatry and even less for emergency situations. Rather, critical discussions of the risk–benefit ratio of each drug compared with potential pharmacological and non-pharmacological alternatives are required.

The most frequently recommended PRISCUS-PIMs in our study were oxazepam > 60 mg/d, lorazepam > 2 mg/d, and haloperidol > 2 mg/d, whereas the most frequently prescribed drugs listed as suitable therapeutic alternatives to PIMs were pipamperone, risperidone, and lorazepam ≤ 2 mg/d. Comparable studies in the inpatient gerontopsychiatric setting also found benzodiazepines (lorazepam > 2 mg/d, diazepam) and antipsychotics (haloperidol > 2 mg/d and olanzapine > 10 mg/d) to be the most frequently prescribed PRISCUS-PIMs.^11,17^ Studies examining the prescribing characteristics of PIMs in the ED detected diphenhydramine, ketorolac, metoclopramide, diazepam, and lorazepam, among others, as the most commonly prescribed PIMs.^21,25,26^ The most frequently prescribed FORTA C drugs in our analysis were pipamperone, risperidone, and oxazepam, while haloperidol and oxazepam accounted for most of FORTA D drugs.

Therefore, possible alternatives for sedating drugs and antipsychotics in the ED should be critically discussed. In our study population, dementia, delirium, and depression represented the most common psychiatric disorders. Possible psychiatric emergency scenarios in the context of these disorders include agitation, aggressiveness, and acute suicidality,^27^ as we also detected in our study. Benzodiazepines or low-potency antipsychotics are particularly suitable for sedation in the acute setting. Lorazepam and oxazepam were among the most frequently prescribed drugs in our collective. Their use was categorized as unfavorable or to be avoided by the FORTA classification, whereas classification as PIMs according to the PRISCUS list depends on their cumulative daily dose.

While the use of oxazepam as current standard therapy for the prophylaxis of alcohol withdrawal symptoms is difficult to avoid even for geriatric psychiatric patients, the use of lorazepam may be considered rather critically in view of its dependence potential, increased risk of falls, and delirogenic potential.^28,29^ For sedation and anxiolysis in psychiatric emergency situations in elderly patients, pipamperone appears to be a suitable alternative.^30,31^ In contrast to other low-potency antipsychotics such as promethazine or levomepromazine, pipamperone has a significantly lower risk of extrapyramidal motor disturbances and fewer anticholinergic side effects.^30,31^ Similarly, recommendations of risperidone in the ED setting may be characterized as favorable, considering risperidone’s low anticholinergic side-effect profile and its proven efficacy in delirium and against dementia-related behavioral disturbances.^32,33^ Haloperidol in age-adapted doses is also suitable in cases of acute agitation or psychosis in the ED, but should not be recommended for long-term use in geriatric patients in view of its numerous side effects.^33^

Avoidance of DDIs is a target of rational drug therapy in elderly patients.^34^ In our study, even though the number of drugs increased significantly, the number of DDIs remained unchanged following psychiatric consultation. In previous studies, the number of DDIs differed significantly, depending on the study design and the interaction program used. The proportion of patients in the ED with at least one DDI in their medication was reported in other studies to be 39 % and 48 %, respectively, but did not refer exclusively to elderly patients.^8,35^ In our study, 43.3 % of all patients had at least one DDI in their medication list before psychiatric consultation and 50 % thereafter. The mean number of DDIs per patient was 1.2 before and 1.3 after psychiatric consultation, a non-significant difference. This appears comparable to the aforementioned studies and does not suggest any specific risks arising from the pharmacological recommendations made by the consultation service.^8,35^

In summary, the proportion of newly recommended drugs listed as PIMs differed substantially depending on the applied PIM classification system. The FORTA classification declared the proportion of PIMs markedly higher than the PRISCUS list, which was due in particular to the contrasting categorization of two commonly prescribed drugs, that is, pipamperone and risperidone. In general, the prescription of psychotropic PIMs can hardly be avoided altogether in the ED. Instead, a list of recommendations specifically designed for this purpose would be necessary for rational evaluation of their use. In general, the fact that many drugs designated as suitable therapeutic alternatives to PIMs according to the PRISCUS list were recommended can be perceived as an indicator of adequate medication safety within the work of the psychiatric consultation service. Significantly more recommendations referred to prescriptions of new drugs compared to drug tapering or discontinuation, suggesting potential for optimization. The number of medications increased significantly following the recommendations of the psychiatric consultation service, potentially contributing to polypharmacy in the geriatric patient population and indirectly increasing the risk of ADRs.^9^ Therefore, it should be stressed that a critical evaluation of the existing medication should always be conducted by psychiatric consultation service physicians. Thus, the number of DDIs in somatically seriously ill patients with psychiatric comorbidity could be reduced. Moreover, a large number of DDIs in our study population were associated with increased bleeding risk or risk of central nervous system depressant effects, which warrants appropriate monitoring of patients.

Our study clearly has limitations. The main limitation is the retrospective and monocentric design and the relatively small sample size. Of note, many patients seen by the psychiatric consultation service (1,044 of 1,263 patients) were below 65 years and therefore did not meet the inclusion criteria of our study. Furthermore, our analysis was based on data from a tertiary care university hospital, making it difficult to generalize our results to peripheral hospitals. The PIM lists applied in our study have only been validated for Germany. Examples of PIM lists used in other countries comprise the Beers criteria (United States) and the Laroche list (France).^36,37^ Our results may therefore be not completely transferable to countries which preferentially use PIM lists other than the PRISCUS list and FORTA classification.

Our study investigated potential risk factors for the development of ADRs, which only allowed an indirect evaluation of medication safety. Prospective multicentric studies are needed in the future to better characterize the influence of psychiatric consultation services on patient-related outcomes.

## Appendix

### Abbreviations

ADR: Adverse drug reaction
AiD: Arzneimittel-Informations-Dienste (Drug Information Services)
DDI: Drug–drug interaction
ED: Emergency Department
FORTA: Fit fOR The Aged
ICD-10: International Statistical Classification of Diseases and Related Health Problems 10^th^ Revision
PIM: Potentially inappropriate medication (for older people)
PRISCUS: Latin for ancient, venerable.
